# Editorials: Dual Reflections on Telerehabilitation

**DOI:** 10.63144/ijt.2026.6770

**Published:** 2026-06-01

**Authors:** Ellen R. Cohn, Kyrylo Malakhov

**Affiliations:** 1 Department of Communication, University of Pittsburgh; University of Maryland Global Campus; and McGowan Institute of Regenerative Medicine; 2 Microprocessor Technology Lab/Department, V.M. Glushkov Institute of Cybernetics of the National Academy of Sciences of Ukraine

**Keywords:** Artificial intelligence, Computer science, Digital health, Generative artificial intelligence, Human digital twins, Research integrity, Rehabilitation medicine, Tele-AAC, Telerehabilitation

## Abstract

Two complementary editorials are presented by two IJT editors. The first editorial reflects upon aspects of the relationship between Artificial Intelligence (AI) and telerehabilitation and points out that current AI terminology, in which AI is employed as “an umbrella term” can be imprecise and confusing. The second editorial observes that telerehabilitation is now entering a new stage of development in which digital health, computer science, artificial intelligence, and rehabilitation medicine are becoming increasingly integrated. The future use of Human Digital Twins as dynamic, patient-centered digital representations will enable individualized assessment, rehabilitation planning, monitoring of patient trajectories, and decision support across distributed care environments.

## AI for Telerehabilitation Is Not the Same as AI for War

### A Note from IJT Co-Editor, Ellen R. Cohn, PhD

The *International Journal of Telerehabilitation* will reach its twenty-year mark in 2028. The types and uses of technology have advanced at a dizzying speed over the life of this journal, although their integration into the conduct of telerehabilitation has been more gradual. It is naïve to believe that any pronouncements made in this Editorial will be valid in a month, in two years, or in the latter part of the next decade.

How does artificial intelligence (AI) employed for telerehabilitation differ from AI used in the conduct of war, or even autonomous vehicles? Generative AI tools for telerehabilitation currently require human oversight for prescriptive clinical use and for authorization of reimbursement in the USA.

The current AI terminology can be imprecise and confusing. In 2026, the term “artificial intelligence” carries both positive and negative connotations. Many acknowledge the medical benefits of AI. There is also the fear that future uses of AI could irreparably alter life as we know it, via the autonomous deployment of weapons and the unchecked development of AI-enabled social engineering capabilities.

Attention to the power of terminology has precedent. Telehealth for rehabilitation has spawned terms that denote positive, specific uses (i.e., tele-AAC, telerehabilitation, tele-speech, etc.). Might the same be helpful to delineate AI use that is exclusive to telerehabilitation? The term AI-telerehabilitation, or AI-telerehab, is one option for assigning a more specific meaning to the use of AI in telerehabilitation.

The editors of this journal have been discussing the acceptable uses of AI in literature. A policy about AI use for IJT articles now seems needed. Yet, too much specificity risks “dating” the journal because AI capabilities and societal expectations will likely evolve. We readily agree on two principles.

First, AI should not be used as a substitute for authorship, scholarly judgment, or original intellectual contribution. This is distinct from grammar checking, language polishing, formatting support, or other limited editorial assistance. The undisclosed use of AI to generate portions or all of an article can create serious risks, including inaccurate attribution, fabricated content, and intentional or unintentional plagiarism. This can even cause unknowing plagiarism. Consider the example published in the New York Times Book Review by book review author Alex Preston who used AI to write portions of a review. The content AI supplied had previously been published in The Guardian by a different author. The AI tool did not alert Preston to the original source, and the New York Times published an embarrassing retraction (Loffhagen, 2026). This was a foreseeable risk because generative AI systems may reproduce, paraphrase, or recombine patterns from previously available textual material without reliably identifying the original source. For IJT submissions, corresponding authors and co-authors must be vigilant and determine whether any AI tool was used to generate, revise, or materially shape manuscript text, and whether such use has been properly disclosed.

A second principle that IJT editors readily agree upon is that the use of generative AI must be transparently disclosed and, when AI-generated output is quoted, paraphrased, or materially relied upon, appropriately cited. The American Psychological Association offers a schema for attribution: https://apastyle.apa.org/blog/cite-generative-ai-references.

Finally, we emphasize that the relationships between a journal, its authors, and its reviewers must be predicated on TRUST: that submitted content is original, that authors remain fully responsible for the work, and that any use of AI is disclosed transparently.

Reference

LoffhagenE

2026
March
31
The New York Times drops freelance journalist who used AI to write book review
The Guardian

https://www.theguardian.com/books/2026/mar/31/the-new-york-times-drops-freelance-journalist-who-used-ai-to-write-book-review



## Reflections on Telerehabilitation: New Stages of Development, Human Digital Twins, and Future Needs

### A Note from Senior Associate Editor, Kyrylo Malakhov

Telerehabilitation is now entering a new stage of development in which digital health, computer science, artificial intelligence, and rehabilitation medicine are becoming increasingly integrated. One of the emerging directions is the use of Human Digital Twins as dynamic, patient-centered digital representations that may support individualized assessment, rehabilitation planning, monitoring of patient trajectories, and decision support across distributed care environments. In this context, generative AI systems, including large language models, should not be viewed merely as text-generation tools, but as components of broader intelligent infrastructures that may assist clinicians, patients, caregivers, researchers, and educators when deployed under appropriate human supervision, ethical governance, and clinical validation.

The future of telerehabilitation will require deeper and more systematic integration of computer science into rehabilitation workflows. This includes ontology engineering, knowledge graphs, multimodal data processing, AI-supported clinical documentation, explainable decision-support systems, virtual standardized patients, AI agents, and secure platforms for remote interaction. These technologies should be developed not as replacements for rehabilitation professionals, but as instruments that extend clinical reasoning, improve accessibility, support continuity of care, and enable more precise personalization of rehabilitation programs.

A vital task for the field is the education of a new generation of healthcare professionals prepared to work responsibly within AI-enhanced telerehabilitation ecosystems. Future physicians, rehabilitation physicians, occupational therapists, physical therapists, speech-language pathologists, nurses, and allied health professionals will need not only clinical expertise, but also practical literacy in digital health, AI safety, data ethics, human-computer interaction, and remote care models. The training of such professionals is essential if telerehabilitation is to evolve from emergency or supplementary remote care into a mature, evidence-based, patient-centered component of modern healthcare systems.

For IJT, this emerging stage creates a distinct responsibility: to support rigorous, clinically grounded, ethically governed, and technologically informed scholarship on telerehabilitation. The journal can serve as a forum where rehabilitation professionals, computer scientists, health informaticians, educators, and policy stakeholders jointly define evidence-based standards for AI-enhanced remote rehabilitation. Such interdisciplinary work is essential to ensure that new intelligent systems are not only technically advanced, but also clinically meaningful, accessible, safe, transparent, and beneficial for patients.
